# Effects of hypercapnia and NO synthase inhibition in sustained hypoxic pulmonary vasoconstriction

**DOI:** 10.1186/1465-9921-13-7

**Published:** 2012-01-31

**Authors:** Farzaneh Ketabchi, Hossein A Ghofrani, Ralph T Schermuly, Werner Seeger, Friedrich Grimminger, Bakytbek Egemnazarov, S Mostafa Shid-Moosavi, Gholam A Dehghani, Norbert Weissmann, Natascha Sommer

**Affiliations:** 1 School of Medicine, Shiraz University of Medical Sciences, Shiraz, Iran; 2 Justus-Liebig-University Giessen, University of Giessen & Marburg Lung Center (UGMLC), Excellence Cluster Cardio-Pulmonary System (ECCPS), Medical Clinic II/IV/V, Aulweg 130, 35392 Giessen, Germany

**Keywords:** hypoxia, hypercapnia, acidosis, nitric oxide, hypoxic pulmonary vasoconstriction

## Abstract

**Background:**

Acute respiratory disorders may lead to sustained alveolar hypoxia with hypercapnia resulting in impaired pulmonary gas exchange. Hypoxic pulmonary vasoconstriction (HPV) optimizes gas exchange during local acute (0-30 min), as well as sustained (> 30 min) hypoxia by matching blood perfusion to alveolar ventilation. Hypercapnia with acidosis improves pulmonary gas exchange in repetitive conditions of acute hypoxia by potentiating HPV and preventing pulmonary endothelial dysfunction. This study investigated, if the beneficial effects of hypercapnia with acidosis are preserved during sustained hypoxia as it occurs, e.g in permissive hypercapnic ventilation in intensive care units. Furthermore, the effects of NO synthase inhibitors under such conditions were examined.

**Method:**

We employed isolated perfused and ventilated rabbit lungs to determine the influence of hypercapnia with or without acidosis (pH corrected with sodium bicarbonate), and inhibitors of endothelial as well as inducible NO synthase on acute or sustained HPV (180 min) and endothelial permeability.

**Results:**

In hypercapnic acidosis, HPV was intensified in sustained hypoxia, in contrast to hypercapnia without acidosis when HPV was amplified during both phases. L-N^G^-Nitroarginine (L-NNA), a non-selective NO synthase inhibitor, enhanced acute as well as sustained HPV under all conditions, however, the amplification of sustained HPV induced by hypercapnia with or without acidosis compared to normocapnia disappeared. In contrast 1400 W, a selective inhibitor of inducible NO synthase (iNOS), decreased HPV in normocapnia and hypercapnia without acidosis at late time points of sustained HPV and selectively reversed the amplification of sustained HPV during hypercapnia without acidosis. Hypoxic hypercapnia without acidosis increased capillary filtration coefficient (*Kfc*). This increase disappeared after administration of 1400 W.

**Conclusion:**

Hypercapnia with and without acidosis increased HPV during conditions of sustained hypoxia. The increase of sustained HPV and endothelial permeability in hypoxic hypercapnia without acidosis was iNOS dependent.

## Background

Acute and chronic respiratory disorders show a high incidence and a high mortality rate of 40-60% worldwide [1-3]. Acute respiratory dysfunctions as occurring in intensive care under conditions like the adult respiratory distress syndrome, lung edema or pneumonia, as well as acute exacerbations of chronic obstructive lung disease or acute neuromuscular damage may induce local or global alveolar hypoxia and hypercapnia. Moreover, hypercapnia is a common condition in the therapeutic approach of permissive hypercapnic ventilation for the treatment of patients with acute lung injury [1]. More than six decades ago it has been concluded by von Euler and Liljestrand that alveolar hypoxia induces HPV for ventilation-perfusion matching in the lung [4]. HPV is the physiological response of precapillary vessels exposed to acute (0-30 min), as well as sustained alveolar hypoxia (> 30 min), in order to improve pulmonary gas exchange under conditions of local hypoxia [5]. Sustained HPV may result in the development of acute pulmonary hypertension under conditions of general alveolar hypoxia. However, it is still controversial how hypercapnia or acidosis affects pulmonary vascular tone and hypoxic vasconstriction. Whereas evidence exists that metabolic acidosis increases pulmonary arterial pressure (PAP), there are inconsistent findings as to whether hypercapnic acidosis has any effect on pulmonary arterial pressure [6-8]. Some reports have pointed out that in isolated lung preparations hypercapnia with normal pH increases, whereas others suggested that it does not change pulmonary vascular tone [9-12].

We recently showed, that hypercapnic acidosis amplified the acute phase of HPV (lasting up to 10 minutes) and improved ventilation-perfusion matching [13], but it is still unknown, if this is true in pathological conditions of sustained hypoxia lasting much more than minutes and at least several hours. In this regard it is important to mention that the acute phase and the sustained phase of HPV are suggested to be regulated, at least in part, by different mechanisms [13,14]. In addition, the sustained phase may be clinically more relevant, as the conditions of repiratory dysfunction, as detailed above, can lead to conditions of prolonged hypoxia, lasting longer than several minutes [15]. Besides a possible deterioration of ventilation-perfusion matching, disturbance of pulmonary gas exchange might be aggravated by development of pulmonary edema during exposure to prolonged hypoxia with hypercapnia.

Nitric oxide (NO) is known as an important modulator of HPV. However, its detailed role for the effects of hypercapnia on HPV is unknown [16,17]. Both, a decrease of NO production during hypercapnia [18-20], as well as an increase in NO production [21-23] has been shown. It was also suggested that the NO production does not change or does not contribute to the regulation of pulmonary vascular tone at above conditions [12,24]. Particularly, 1) the general role of NO synthases (NOS), 2) a differentiation between the role of endothelial NOS (eNOS) and inducible NOS (iNOS), as well as 3) effects of NO synthase inhibitors (L-NNA and 1400 W, respectively) on sustained HPV under hypercapnia have not been investigated yet.

Against this background, we 1) compared the effects of hypercapnia with or without acidosis on HPV and endothelial permeability under conditions of acute and sustained hypoxia and 2) deciphered the potential therapeutic effects of NO inhibitors in such conditions. Our investigations were performed in isolated, perfused and ventilated rabbit lungs, a model that allows detailed quantification of pulmonary hemodynamic and biochemical events without interference with systemic regulatory mechanisms.

## Methods

### Lung isolation, perfusion, and ventilation

Animal experiments were approved by the local authorities (Regierungspräsidium Giessen) in Germany. The model of isolated perfused rabbit lungs has been described previously [25]. Briefly, pathogen free male rabbits (body weight 2.8-3.8 kg) were deeply anaesthetized with an i.v. application of ketamine (30-50 mg/kg) and xylazine (6-10 mg/kg) and treated with the anticoagulant heparin (1000 U/kg body weight). The trachea was cannulated and animals were ventilated with room air (tidal volume 30 ml, frequency 30 strokes/min). The lungs were perfused with Krebs-Henseleit solution (perfusate) through the pulmonary artery. After rinsing the lungs with 1 liter of the perfusate for washout of the blood, the perfusion circuit was closed for circulation and the start of the experiments. Meanwhile, the flow rate was slowly increased from 20 to 150 ml/min, and concomitantly the left atrial pressure was set at 2 mmHg. A positive end expiratory pressure of 2 cm H_2_O was chosen for prevention of regional alveolar collapse.

The isolated perfused lung was placed in a temperature equilibrated housing chamber and freely suspended from a force transducer (Hottinger Baldwin, Germany) for continuous monitoring of organ weight. The whole system (perfusate, reservoirs, tubing, and housing chamber) was heated to 38.5°C. Pressures in the pulmonary artery, left atrium, and trachea were continuously registered. All lungs included in the study 1) exhibited a homogeneous white appearance with no signs of hemostasis, edema, or atelectasis, 2) revealed a constant mean pulmonary artery and peak ventilation pressures in the normal range, and 3) were isogravimetric during the first 20 min of steady state period. Because flow-rate of the perfusate was constant, changes in pulmonary artery pressure are proportional to pulmonary vascular resistance.

### Composition of ventilatory gas and perfusate

Four different gas mixtures were employed for ventilation of the lung during different experimental conditions (Gas Mixing Chamber, KM 60-3/6MESO, Witt, Witten, FRG, Germany): 1) normoxia plus normocapnia: 21.0% O_2_, 5.3% CO_2 _balanced with N_2_, 2) normoxia plus hypercapnia: 21.0% O_2_, 11.0% CO_2_, balanced with N_2_, 3) hypoxia plus normocapnia: 3.0% O_2_, 5.3% CO_2 _balanced with N_2_, 4) hypoxia plus hypercapnia: 3.0% O_2_, 11.0% CO_2 _balanced with N_2_. The perfusate used for the study contained 120.0 mM NaCl, 1.1 mM K_2_HPO_4_, 1.3 mM MgCl_2_, 4.3 mM KCl, 2.4 mM CaCl_2_, 13.3 mM glucose, 50 g hydroxy-ethyl-amylopectin (MW: 200000). In non-acidotic experiments pH was adjusted with NaHCO_3 _(sodium-bicarbonate 1 M 8.4% [Serag-Wiessner KG]) to a physiological range of 7.35-7.40.

In all groups, PO_2_, PCO_2 _and pH of the perfusate were measured with a gas analyzer 10 min after starting the experiment and then, 10 min after initiating experimental conditions. Table 1 shows the values taken 10 min after initiating each experimental condition. The values of PO_2_, PCO_2 _and pH remained stable during the time course of each experiment.

**Table 1:** pH, PCO_2_, PO_2 _and [HCO_3_^-^] in the perfusate during the different experimental conditions

Parameters	Normoxic normocapnia	Normoxic hypercapnic acidosis	Normoxic hypercapnia w/o acidosis	Hypoxic normocapnia	Hypoxic hypercapnic acidosis	Hypoxic hypercapnia w/o acidosis
PO_2_ (mmHg)	158 ± 2	162 ± 1	165 ± 0	51 ± 7 *	62 ± 1 *	64 ± 1 *

PCO_2_ (mmHg)	37 ± 0	71 ± 1 *	72 ± 0 *	37 ± 0	72 ± 0 *	72 ± 1 *

pH	7.38 ± 0.00	7.08 ± 0.00 *	7.38 ± 0.00	7.36 ± 0.00	7.09 ± 0.00 *	7.38 ± 0.00

[HCO3^-^] (mmol/L)	21 ± 0	21 ± 0	42 ± 0 *	20 ± 0	21 ± 0	41 ± 0 *

Data are mean ± SEM for n = 5-8 experiments each, derived 10 min after initiating each experimental condition. No difference of the respective parameters could be detected during the course of the experiment (data not shown). In hypercapnia with normal pH, pH was corrected to the given values by the addition of sodium bicarbonate.

* indicates significant differences as compared to normoxia or normocapnia.

### Gravimetric determination of capillary filtration coefficient

Capillary filtration coefficient (K_fc_) was quantified to assess changes in capillary permeability by a hydrostatic challenge. It was determined gravimetrically from the slope of the lung weight-gain curve induced by a stepwise elevation of venous pressure by 5.5 mmHg for 8 minutes, as described previously [26].

### Evaluation of exhaled NO

The technique of measurement of exhaled NO levels has been described previously [17]. In brief, an aliquot of the mixed expired gas was continuously forwarded to a chemiluminescence NO analyzer (280 NOA; Sievers Instruments, Boulder, CO) to measure exhaled NO concentration.

### Study protocol

Forty minutes after the establishment of normoxic normocapnic ventilation, the first hydrostatic challenge was performed by elevating pulmonary venous outflow pressure from 2.0 to 7.5 mmHg for 8 min. Fifteen minutes later, a 10 min period of hypoxic ventilation was performed to determine the response to alveolar hypoxic normocapnia, in order to ensure comparable vasoreactive potential of the pulmonary vasculature of the individual lungs. Subsequently, hypoxic normocapnic ventilation of the lung was changed to 15 min normoxic normocapnic ventilation. Thereafter, each lung was randomly ventilated for 180 min (0-30 min: acute phase, 30-180 min: sustained phase) with different gas mixtures as described above (please refer to: experimental conditions) followed by 10 min normoxic normocapnic ventilation (180-190 min: recovery phase). Thirty minutes later, a second K_fc _measurement was performed. In the pharmocologically treated groups, L-NNA (200 μM) or 1400 W (2 μM) was added to the perfusate 10 min prior to the onset of the first hydrostatic challenge.

### Statistical Analysis

Data are given as mean ± SEM. Analysis of variance (ANOVA) with the Student-Newman-Keuls (SNK) post hoc test was used for comparison of more than two groups. For comparison of the values during the time course of one group, one way ANOVA for repeated measurements with SNK post hoc analysis was used. For the comparison of two groups a Student's t-test was applied. Significance was assumed for P < 0.05.

## Results

### Effect of hypercapnia with and without acidosis on HPV in the presence and the absence of L-NNA and 1400 W

**Figure 1:**
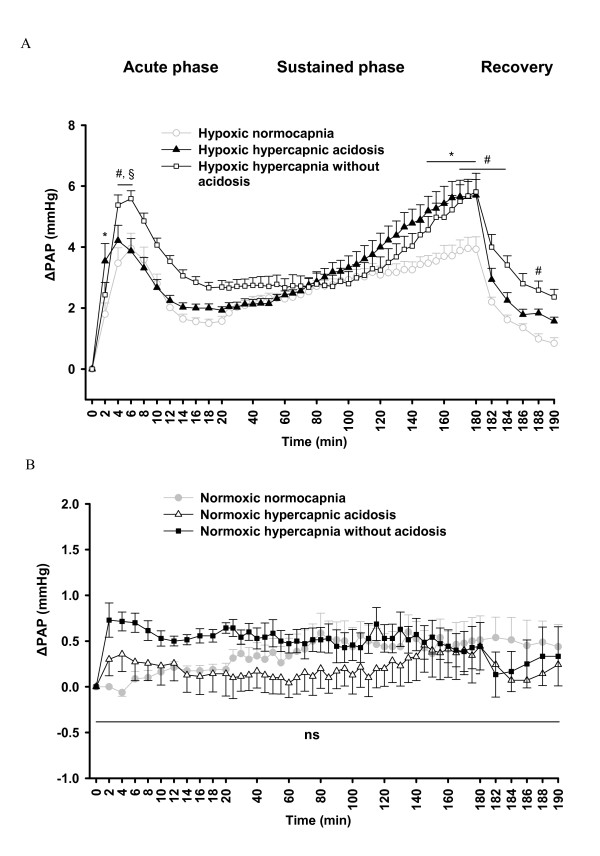
**Effects of hypercapnia with or without acidosis on changes in pulmonary arterial pressure (ΔPAP) during hypoxia, and normoxia**. **A**. Changes of PAP in hypoxic normocapnia (n = 7), hypoxic hypercapnic acidosis (n = 7), and hypoxic hypercapnia without acidosis (n = 8) during 180 min of hypoxic ventilation, followed by 10 min ventilation with normoxic normocapnic gas (recovery). ΔPAP: change of PAP referenced to the baseline value at time set at zero. **B**. Changes of PAP in normoxic normocapnia (n = 8), normoxic hypercapnic acidosis (n = 8) and normoxic hypercapnia without acidosis (n = 7) during 190 min normoxic ventilation. ΔPAP: change of PAP referenced to the value at time set at zero. All data are mean **± **SEM. *, significant difference (P < 0.05) between hypoxic normocapnia and hypoxic hypercapnic acidosis. #, significant difference (P < 0.05) between hypoxic normocapnia and hypoxic hypercapnia without acidosis. §, significant difference (P < 0.05) between hypoxic hypercapnic acidosis and hypoxic hypercapnia without acidosis.

Mean normoxic normocapnic pulmonary artery pressure (PAP) values prior to the onset of hypoxia were 10.0 ± 1.5 mmHg (n = 7). During hypoxic normocapnia, the initial increase of PAP (ΔPAP) reached the maximum value of 4.0 ± 0.5 mmHg at 6 min after induction of hypoxia ("acute phase"), was followed by a decline to a ΔPAP of 1.6 ± 0.2 mmHg at minute 14, and then remained stable up to minute 18. Subsequently, in the sustained phase of HPV, ΔPAP was gradually elevated reaching a maximum value of 4.0 ± 0.4 mmHg at 175 min ("sustained phase", Figure 1A).

Mean normoxic normocapnic PAP values before the onset of hypoxic hypercapnic acidosis were 10.1 ± 0.5 mmHg (n = 7). Ventilation with hypoxic hypercapnic acidosis resulted in an enhancement of the sustained phase of HPV compared to normocapnia, ΔPAP ranged from 5.2 ± 0.5 mmHg at minute 150 to 5.7 ± 0.5 mmHg at minute 180, whereas the maximal strength of HPV during the acute phase (ΔPAP 4.2 ± 0.5 mmHg at minute 4) was unchanged, but showed a faster rise (3.5 ± 0.6 mmHg during hypoxic hypercapnic acidosis vs. 1.8 ± 0.4 mmHg during hypoxic normocapnia at minute 2) (Figure 1A).

Mean normoxic normocapnic PAP values before the onset of hypoxic hypercapnia without acidosis were 9.9 ± 0.4 mmHg (n = 7). Correction of acidosis with sodium bicarbonate in the hypoxic hypercapnia without acidosis, resulted in increased HPV during the acute as well as the sustained phase compared to hypoxic normocapnia. The initial strong vasoconstrictive response (maximum value of 5.6 ± 0.3 mmHg at 6 min of exposure) declined to 2.7 ± 0.2 mmHg at 18 min. There was no significant difference between the values of ΔPAP during the sustained phase of the maneuvers in the hypoxic hypercapnic with or without acidosis groups (Figure 1A).

There were no significant changes in normoxic pressure during the course of the experiments in normoxic normocapnia or normoxic hypercapnia with or without acidosis groups (Figure 1B).

**Figure 2:**
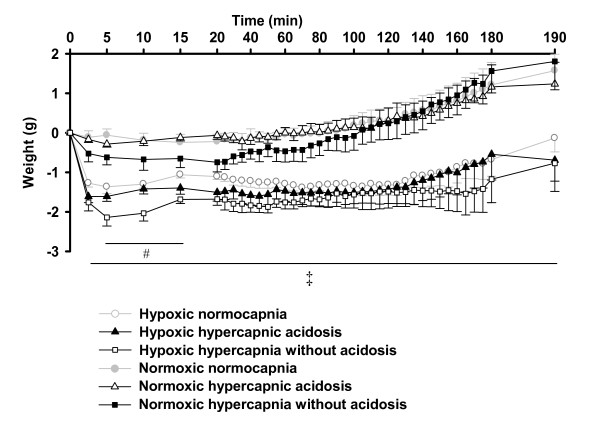
**Effects of hypercapnia with or without acidosis on changes in lung weight during hypoxia, and normoxia**. Lung weight changes of the respective experiments of figure 1. All data are mean **± **SEM. #, significant difference (P < 0.05) between hypoxic normocapnia and hypoxic hypercapnia without acidosis. ‡, significant difference (P < 0.05) between hypoxic groups and normoxic normocapnia.

Hypoxia led to a reduced lung weight during all three conditions compared to normoxia. In hypoxia, the decremental changes of lung weight during 5-15 min were larger in the hypoxic hypercapnia without acidosis group than the hypoxic normocapnic group, but no difference was detectable between hypoxic normocapnia and hypoxic hypercapnic acidosis (Figure 2).

**Figure 3:**
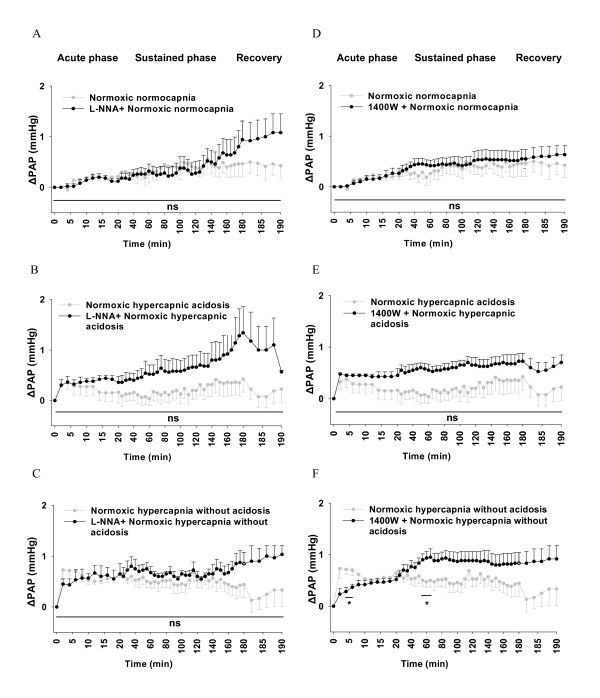
**Effects of L-N^G^-Nitroarginine (L-NNA) and N-([3-(Aminomethyl) phenyl] methyl) ethanimidamide dihydrochloride (1400 W) on pulmonary vascular tone during normoxic ventilation**. Normoxic ΔPAP values in the absence of L-NNA and 1400 W are identical to those depicted in Figure 1. **A**. L-NNA-treated normoxic normocapnia (n = 5), **B**. L-NNA-treated normoxic hypercapnic acidosis (n = 5), **C**. L-NNA-treated normoxic hypercapnia without acidosis (n = 4), **D**. 1400 W-treated normoxic normocapnia (n = 6), **E**. 1400 W-treated normoxic hypercapnic acidosis (n = 4), **F**. 1400 W-treated normoxic hypercapnia without acidosis (n = 6) compared to **ΔPAP **from lungs in the absence of L-NNA or 1400 W during 190 min of normoxic ventilation. ΔPAP: change of PAP referenced to the value at time set at zero. Data are mean ± SEM. *, significant difference (P < 0.05) between treated and untreated groups.

In the presence of L-NNA, no significant changes of pulmonary artery pressure were detectable under normoxic ventilation, although a tendency towards higher values at later time points was evident (Figure 3A, B, C). Treatment with 1400 W did neither change normoxic normocapnic PAP nor PAP during normoxic hypercapnic acidosis compared to the untreated group (Figure 3D, E). However, during normoxic hypercapnia without acidosis 1400 W induced a significant PAP decrease at min 4-6 and an increase at min 55-65 compared to lungs in the absence of 1400 W (Figure 3F).

**Figure 4:**
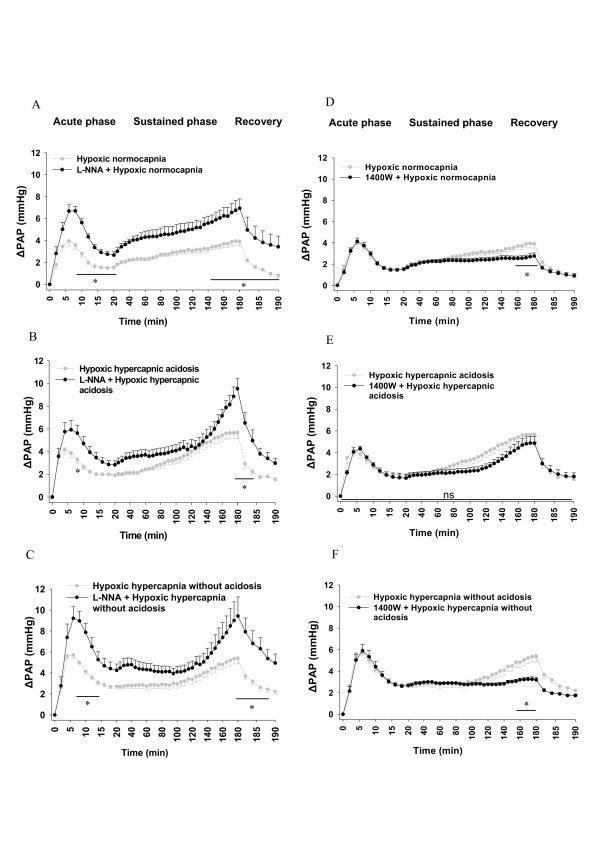
**Effects of L-NNA and 1400 W on the strength of HPV**. Hypoxic ΔPAP values in the absence of L-NNA and 1400 W are identical to those depicted in Figure 1. **A**. L-NNA-treated hypoxic normocapnia group (n = 5), **B**. L-NNA-treated hypoxic hypercapnic acidosis (n = 6), **C**. L-NNA-treated hypoxic hypercapnia without acidosis (n = 5), **D**. 1400 W-treated hypoxic normocapnia (n = 6), **E**. 1400 W-treated hypoxic hypercapnic acidosis (n = 5), **F**. 1400 W-treated hypoxic hypercapnia without acidosis (n = 5) compared to HPV in the absence of L-NNA or 1400 W during 180 min hypoxic ventilation, followed by 10 min ventilation with normoxic normocapnic gas (recovery). ΔPAP: change of PAP referenced to the value at time set at zero. Data are mean **± **SEM. *, significant difference (P < 0.05) compared to untreated groups.

In the presence of L-NNA, the acute and sustained phase of HPV was increased in all groups (Figure 4A, B, C). In contrast, 1400 W decreased the sustained phase of HPV at late time points in normocapnia and hypoxic hypercapnia without acidosis, but not in hypoxic hypercapnic acidosis compared to the respective controls in the absence of 1400 W (Figure 4D, E, F).

**Figure 5:**
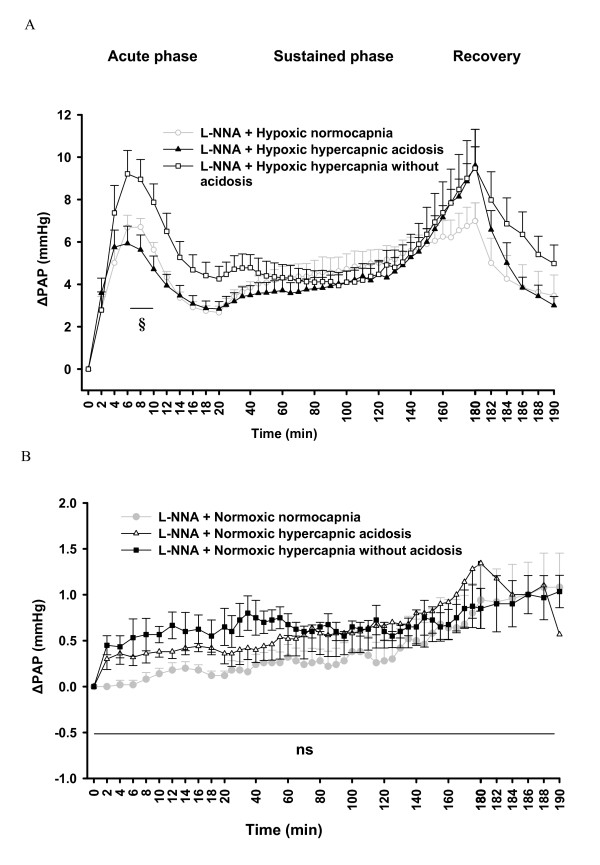
**Effects of L-NNA on the strength of HPV during normocapnia and hypercapnia**. ΔPAP values in the presence of L-NNA are identical to those depicted in Figure 3 A-C and Figure 4 A-C. **A**. Changes in ΔPAP (ΔPAP) during 180 min hypoxic ventilation, followed by 10 min ventilation with normoxic normocapnic gas. ΔPAP: change of PAP referenced to the baseline value at time set at zero. **B**. Changes in PAP (ΔPAP) during 190 min of normoxic ventilation. ΔPAP: change of PAP referenced to the value at time set at zero. All data are mean **± **SEM. §, significant difference (P < 0.05) between hypoxic hypercapnic acidosis and hypoxic hypercapnia without acidosis.

**Figure 6:**
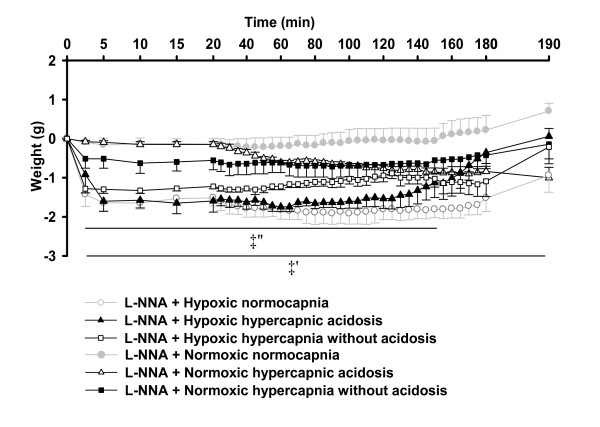
**Effects of L-NNA on lung weight during normocapnia and hypercapnia**. Lung weight changes of the respective experiments of figure 5. Data are mean **± **SEM. ‡', significant difference (P < 0.05) between hypoxic normocapnia and normoxic normocapnia. ‡'', significant difference (P < 0.05) between hypoxic hypercapnia with or without acidosis and normoxic normocapnia.

**Figure 7:**
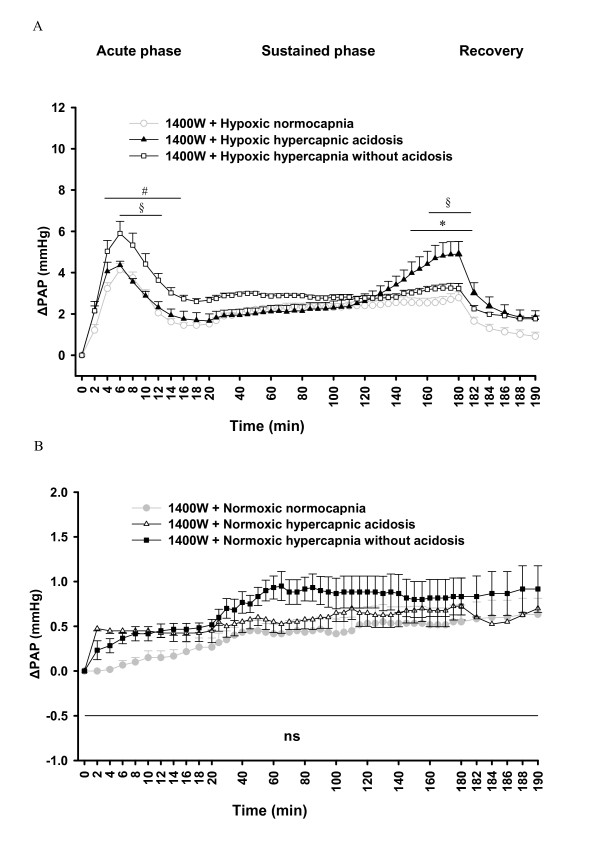
**Effects of 1400 W on the strength of HPV during normocapnia and hypercapnia**. ΔPAP values in the presence of 1400 W are identical to those depicted in Figure 3 D-F and Figure 4 D-F. **A**. Changes in PAP (ΔPAP) during 180 min hypoxic ventilation, followed by 10 min ventilation with normoxic normocapnic gas. ΔPAP: change of PAP referenced to the value at time set at zero. **B**. Changes of PAP (ΔPAP) during 190 min of normoxic ventilation. ΔPAP: change of PAP referenced to the value at time set at zero. All data are mean **± **SEM. *, significant difference (P < 0.05) between hypoxic normocapnia and hypoxic hypercapnic acidosis. #, significant difference (P < 0.05) between hypoxic normocapnia and hypoxic hypercapnia without acidosis. §, significant difference (P < 0.05) between hypoxic hypercapnic acidosis and hypoxic hypercapnia without acidosis.

**Figure 8:**
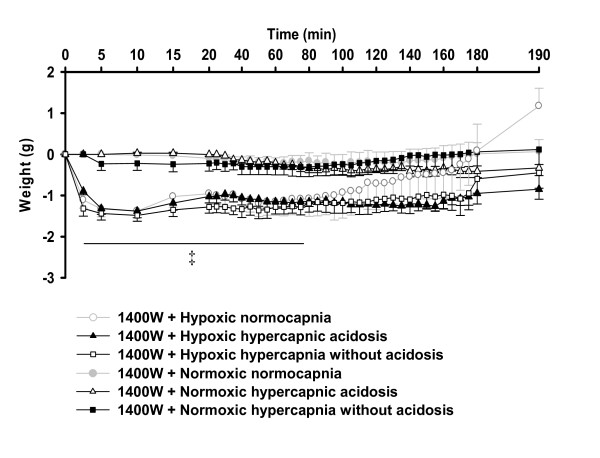
**Effects of 1400 W on lung weight during normocapnia and hypercapnia**. Lung weight changes of the respective experiments of figure 7. Data are mean **± **SEM. ‡, significant difference (P < 0.05) between hypoxic groups and normoxic normocapnia.

For better visualization of the effect of hypercapnia on HPV in the presence of L-NNA or 1400 W, ΔPAP values of the normocapnic and hypercapnic groups from Figure 3 and 4 are displayed in Figure 5A/B and 7A/B in a direct comparison. In the presence of L-NNA the effects of hypercapnia on acute HPV were similar (Figure 5A) as in the absence of L-NNA (Figure 1A) but the increase in sustained hypoxia was no longer significant, compared to hypoxic normocapnia. Normoxic values were not different between the groups in presence of L-NNA (Figure 5B). Moreover, during L-NNA treatment the lung weights showed no difference between hypoxic groups (Figure 6). In presence of 1400 W, ΔPAP was increased in sustained hypoxic hypercapnic acidosis at 150-182 min, but not in hypercapnia without acidosis compared to hypoxic normocapnia. Acute HPV was still increased (minutes 4-16) during hypoxic hypercapnia without acidosis compared to hypoxic normocapnia, similar to the effect in the absence of 1400 W (Figure 7A). Normoxic values were not different between the groups in presence of 1400 W (Figure 7B). Moreover, during 1400 W treatment the lung weights showed no difference between hypoxic groups (Figure 8).

### Effect of hypoxia, hypercapnia with and without acidosis on NO production

**Figure 9:**
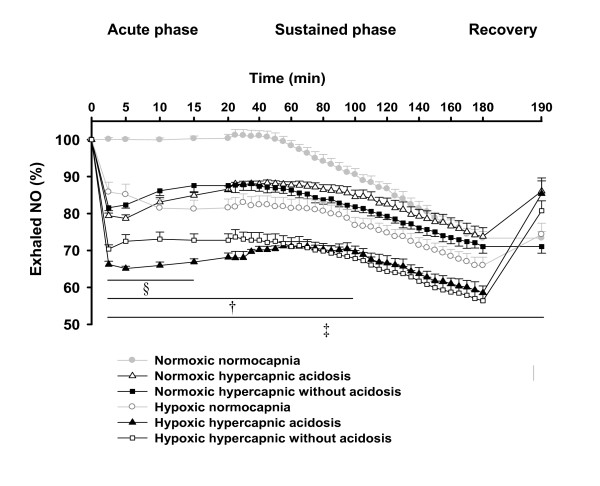
**Effect of hypercapnia with and without acidosis on exhaled NO**. Data are means ± SEM. n = 5-7 each. §, significant difference (P < 0.05) between hypoxic hypercapnic acidosis and hypoxic hypercapnia without acidosis. ‡, significant difference (P < 0.05) between hypoxic groups and normoxic normocapnia. †, significant difference (P < 0.05) between normoxic hypercapnia with or without acidosis compared to normoxic normocapnia. % = percentage of NO referenced to the baseline value at time set at zero.

In normoxic normocapnia, exhaled NO decreased gradually reaching a minimum of 73 ± 2% of its baseline value at 180 min of normoxic ventilation. In normoxic hypercapnia with or without acidosis, exhaled NO declined immediately to 79 ± 1% and 82 ± 1% of the respective baseline values within 2 min of normoxic hypercapnic ventilation, respectively and remained significantly lower than the normocapnic group up to 100 min of the experiment. In hypoxic normocapnia, the exhaled NO rapidly decreased to 86 ± 3% of its baseline value within 2 min of hypoxic ventilation, reached 66 ± 3% of its baseline value at 180 min of hypoxic ventilation and was significantly different from normoxic normocapnia during the entire period of hypoxic ventilation. In hypoxic hypercapnia with or without acidosis exhaled NO dropped to 66 ± 1% and 71 ± 1% of their baseline values, respectively, within 2 min of the experiment. The exhaled NO values during the entire period of the experiment in both hypoxic hypercapnia groups were significantly lower than those of the normoxic groups, as well as compared to hypoxic normocapnia (Figure 9).

### Effect of hypoxia, hypercapnia with and without acidosis on endothelial permeability in the presence and the absence of L-NNA and 1400 W

**Figure 10:**
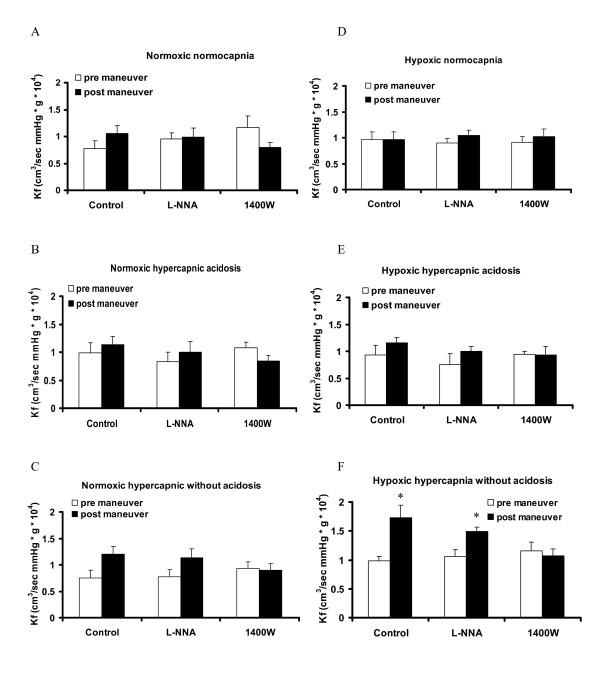
**Effect of hypercapnia with or without acidosis, L-NNA and 1400 W on endothelial permeability**. **A-C**: Normoxic ventilation. **D-F**: Hypoxic ventilation. Endothelial permeability was assessed by measurement of the capillary filtration coefficient (K_fc_) prior (pre maneuver) and after sustained hypoxia (post maneuver). Data are mean **± **SEM. n = 3-7 each. *, significant difference (P < 0.05) between pre maneuver and post maneuver values.

The post-hypoxia K_fc _value was significantly higher in hypoxic hypercapnia without acidosis than in hypoxic normocapnia and normoxic normocapnia, as well as compared to its own pre-hypoxic value (Figure 10). This effect was no longer detectable in the presence of 1400 W, but was still detectable in the presence of L-NNA (Figure 10F). Post-hypoxic K_fc _values of the other groups were not different between each other and from their own pre-hypoxic values (Figure 10A, B, C, D, E).

## Discussion

Although it is known for decades that alveolar hypoxia induces HPV, the effects of hypercapnia on pulmonary vascular tone and capillary permeability under sustained hypoxia, are still not fully elucidated. In addition, the role of NO signaling under such conditions is not fully understood yet. HPV is essential to optimize pulmonary gas exchange during local acute and prolonged hypoxia. As previously shown by our group, hypercapnia was concluded to be beneficial for ventilation-perfusion matching in conditions of repetitive acute hypoxic episodes, each lasting up to 10 minutes. Hypercapnic acidosis improved arterial pO_2_, and augmented HPV without affecting endothelial permeability [13]. In extension of these investigations we now focused on the effects of sustained hypoxic hypercapnia on HPV and pulmonary endothelial permeability. Furthermore, we addressed the role of endothelial and inducible NO synthases in prolonged hypoxic hypercapnia.

Our data revealed that during sustained hypoxia both hypercapnia with and hypercapnia without acidosis increased HPV. However, only hypercapnia without acidosis enhanced acute HPV in the first minutes of the hypoxic ventilation, as shown for short term hypoxia in our previous study [13]. This finding is reminiscent of the effect of hypercapnia on repetitive episodes of short term HPV, when hypercapnia without acidosis increased acute HPV during the first repetitive hypoxic maneuvers, but hypercapnia with acidosis enhanced HPV only in the late episodes of repetitive hypoxic maneuvers [13]. It is also in line with findings that hypercapnic acidosis caused a marked increase in pulmonary vascular resistance during a time period of 60-80 min hypoxia, but not at earlier time points [27]. However, with regard to the effect of hypercapnia on HPV, there are inconsistent findings, with studies differing from our data showing inhibition [28,29] or decrease of HPV [8].

Since, there were no statistically significant alterations in PAP during the entire period of exposure to normoxic hypercapnia, the increased ΔPAP in the sustained phase of the hypoxic hypercapnic groups denotes to the potentiating effect of hypercapnia on the hypoxic response of pulmonary vasculature. In previous studies hypercapnia was reported to induce vasoconstriction in isolated rabbit lungs and rat arteries [10,12,30], vasodilation in isolated rat lungs and arteries [31,32] or to have no effect on pulmonary vascular tone in isolated rat arteries [9,11] in normoxia. There are, however, differences in experimental conditions and protocols that might explain these conflicting results.

Examining the role of NO, our study suggests 1) that NO counteracts the increase of PAP in both phases of HPV, but has no effect on pulmonary vascular tone under normoxic conditions, 2) that the increase of the sustained phase of HPV during hypoxic hypercapnia without acidosis, and partially during hypoxic normocapnia, is related to iNOS, whereas the increase in hypoxic hypercapnia with acidosis is related to eNOS, as during general NOS inhibition the significant increase of sustained HPV caused by hypercapnia with and without acidosis disappeared, whereas such an effect was only true for the iNOS inhibitor during hypercapnia without acidosis. This statement is, with regard to eNOS, however, only valid, if a potential role of nNOS can be neglected as suggested by measurements in nNOS knock-. out mice that showed no alteration of HPV and endothelial dependent or endothelial independent vasoconstriction in isolated lungs [33]. 3) Our data show that iNOS activity, but not eNOS activity is responsible for the increase in endothelial permeability occurring only during hypoxia without acidosis, as only the iNOS inhibitor, but not general NOS inhibition by L-NNA prevented the increase in the capillary filtration coefficient.

The first conclusion is based on the findings, that during normoxia inhibition of NOS by L-NNA did not change the level of vascular tone and inhibition of iNOS showed only a decrease of ΔPAP during normoxic hypercapnia without acidosis at the beginning and increase of ΔPAP at ~60 min of the experiments. In contrast, L-NNA increased PAP during all conditions in hypoxia. As the iNOS inhibitor had not such an effect, most probably eNOS is responsible for this amplification of in acute and sustained HPV. In line with these findings it has been shown that NO is not involved in regulation of normoxic pulmonary vascular tone, neither during normocapnia [34,35] nor during hypercapnia [8,12], although some interspecies variability may exist [34,36]. Thus, NO is involved in the regulation of pulmonary vascular responses to hypoxia as previously shown [5,36].

The second conclusion is based on the findings of differential amplification and inhibition of HPV, by general NOS inhibition with L-NNA and specific iNOS inhibition with 1400 W, respectively. General NOS inhibition increased acute and sustained HPV in all conditions and eliminated the difference between hypoxic normocapnia and hypoxic hypercapnia with and without acidosis in sustained HPV. Specific iNOS inhibition only inhibited the difference between hypoxic normocapnia and hypoxic hypercapnia without acidosis in sustained HPV. Thus, eNOS may be responsible for the amplification of sustained HPV in hypercapnic acidosis, whereas iNOS may be responsible for the amplification of sustained HPV in hypercapnia without acidosis. Acute HPV, however, seems to be potentiated in hypercapnia without acidosis by different mechanisms than NO release. The fact that application of 1400 W did not alter ΔPAP at early time points in hypoxia is consistent with a report of Resta et al. showing that iNOS does not modulate pulmonary vasoconstriction in response to a thromboxane analogue [37]. However, in sustained hypoxia 1400 W decreased ΔPAP in hypoxic normocapnia and hypoxic hypercapnia without acidosis, which may be due to upregulation of iNOS in long term hypoxia, as shown before [37]. Uncoupled iNOS activity may lead to production of reactive oxygen and nitrogen species (ROS/RNS), which are suggested to increase HPV [38]. Hypercapnic acidosis may prevent such effects as sustained HPV was not changed in hypoxic hypercapnic acidosis. Along these lines decreased production of ROS and RNS has been shown in hypercapnic hypoxia [39]. Although we can only speculate about the effects of iNOS inhibition on ΔPAP in normoxic hypercapnia without acidosis, the missing effect at late time points supports the finding of a specific activation of iNOS by hypercapnia without acidosis in hypoxia.

During hypoxia the biphasic increase of ΔPAP is paralleled by a concomitant reduction in exhaled NO, as shown before for pure hypoxia by our laboratories [5]. The more pronounced decrease of NO during hypoxic hypercapnia correlated to increased sustained HPV compared to hypoxic normocapnia. However, elevated acute HPV in hypoxic hypercapnia without acidosis compared to hypercapnic acidosis did not correspond to a different NO release, which emphasizes the importance of other factors than NO transmitting the effects of hypercapnia on acute HPV. In line with this explanation, inhibition of NO production did not potentiate short term HPV under hypercapnic acidosis [22,40].

Our third conclusion is based on the findings that only hypercapnia without acidosis resulted in an increased capillary filtration coefficient (K_fc_) after sustained hypoxia. This effect is in accordance with findings that hypercapnic acidosis did not influence pulmonary capillary permeability in repetitive short term hypoxia [13,41,42] or even decreased edema formation in acute lung injury, whereas buffering of hypercapnia with bicarbonate worsened lung injury [43]. This effect may be related to the acidosis induced by hypercapnia, as metabolic acidosis is known to be protective against ischemia-reperfusion induced lung injury in rat lungs [44]. Hypercapnia, in contrast, has been reported to exert damaging effects in the lung [21,23,45-48]. The increase in K_fc _during hypercapnia without acidosis could be inhibited by 1400 W, but not by L-NNA in our study, being in line with previous studies showing that iNOS inhibition is protective against the cellular damage during hypercapnic acidosis [49]. Additionally, hypercapnia has been reported to induce injury in the fetal rat alveolar epithelial cells through a NO dependent pathway [21]. The increase in K_fc _during hypercapnia without acidosis in repetitive episodes of short term hypoxia, however, could also be inhibited by L-NNA [13], which indicates that sustained hypoxia rather activates iNOS than eNOS. The protective effect of iNOS inhibition on the K_fc _increase in hypercapnia without acidosis can, besides a direct effect of NO on endothelial integrity also (at least in part) be related to the decreased pulmonary artery pressure caused by 1400 W application.

## Conclusion

Our study showed that hypercapnia with and without acidosis has different effects on the acute and sustained phase of HPV. Hypercapnic acidosis may have beneficial effects on ventilation-perfusion matching during prolonged hypoxia by increasing HPV and preserving pulmonary capillary integrity. However, it must also be taken into account that prolonged hypoxic hypercapnia can facilitate right heart failure. As the hypoxia-induced increase in pulmonary vascular resistance is rather low [50,51] this may be mainly important in predispositioned patients, if our findings are transferable to the human situation. Whereas general NOS inhibition increased acute and sustained HPV during normocapnia and hypercapnia with and without acidosis, iNOS inhibition decreased sustained HPV in normocapnia and hypercapnia without acidosis. In addition the increase of endothelial permeability in hypercapnia without acidosis can be attributed to increased iNOS activity.

## Competing interests

The authors declare that they have no competing interests.

## Authors' contributions

FK carried out the experiments of isolated lungs and drafted the manuscript. NS analyzed and interpreted the data and drafted the manuscript. HG made substantial contribution to the conception and design of the study and revised the manuscript. WS made substantial contribution to the conception and design of the study and revised the manuscript. FG made substantial contribution to the conception and design of the study and revised the manuscript. BE participated in the experiments of the isolated lungs and drafted the manuscript. SM made substantial contribution to the conception and design of the study and revised the manuscript. GD made substantial contribution to the conception and design of the study and revised the manuscript. NW made substantial contribution to the conception and design of the study, analyzed and interpreted the data and revised the manuscript. All authors read and approved the final manuscript.
